# Consequences of Secondary Calibrations on Divergence Time Estimates

**DOI:** 10.1371/journal.pone.0148228

**Published:** 2016-01-29

**Authors:** John J. Schenk

**Affiliations:** Department of Biology, Georgia Southern University, Statesboro, Georgia, United States of America; BiK-F Biodiversity and Climate Research Center, GERMANY

## Abstract

Secondary calibrations (calibrations based on the results of previous molecular dating studies) are commonly applied in divergence time analyses in groups that lack fossil data; however, the consequences of applying secondary calibrations in a relaxed-clock approach are not fully understood. I tested whether applying the posterior estimate from a primary study as a prior distribution in a secondary study results in consistent age and uncertainty estimates. I compared age estimates from simulations with 100 randomly replicated secondary trees. On average, the 95% credible intervals of node ages for secondary estimates were significantly younger and narrower than primary estimates. The primary and secondary age estimates were significantly different in 97% of the replicates after Bonferroni corrections. Greater error in magnitude was associated with deeper than shallower nodes, but the opposite was found when standardized by median node age, and a significant positive relationship was determined between the number of tips/age of secondary trees and the total amount of error. When two secondary calibrated nodes were analyzed, estimates remained significantly different, and although the minimum and median estimates were associated with less error, maximum age estimates and credible interval widths had greater error. The shape of the prior also influenced error, in which applying a normal, rather than uniform, prior distribution resulted in greater error. Secondary calibrations, in summary, lead to a false impression of precision and the distribution of age estimates shift away from those that would be inferred by the primary analysis. These results suggest that secondary calibrations should not be applied as the only source of calibration in divergence time analyses that test time-dependent hypotheses until the additional error associated with secondary calibrations is more properly modeled to take into account increased uncertainty in age estimates.

## Introduction

Methods that estimate evolutionary divergence times have come a long way in the 50 years since the molecular clock was introduced by Zuckerkandl and Pauling [1–3]. Although the application of a strict molecular clock was rightfully met with apprehension because many data sets significantly deviated from a constant substitution rate [2], relaxed-clock methods have been shown to be robust in the face of substitution rate heterogeneity [4,5]. Examples of significant methodological advances include local molecular-clocks [6], the penalized-likelihood approach implemented in r8s [7,8], the multigene Bayesian-approach implemented in Multidivtime [9–11], and the Bayesian approach that implements the uncorrelated lognormal (UCLN) model (among others) in Beast [12,13]. The behavior of divergence time estimates in the face of uncertainty and violations of methodological assumptions is also better understood because of studies that have investigated the effects of the placement, number, and distribution of calibrated nodes [14–19], consistency among calibrations [20,21], the effects of the number of loci or number of sites [22,23], DNA substitution model misspecification [24,25], DNA saturation [26], and incomplete taxon sampling [27].

Despite the methodological advances in divergence time estimates, empirical data sets often remain difficult to calibrate because many groups lack fossil data. Common solutions to not having fossil calibrations include applying a substitution rate estimated from close relatives to infer divergence times in a focal group [28,29], and calibrating a node with an age estimate from a previous molecular-dating study that applied a fossil calibration. Such calibration schemes are called secondary calibrations because the primary fossils are not included in age estimates [30]. In 2004, Graur and Martin published a critical assessment of how divergence times were being estimated, in which they lamented against the application of secondary calibrations, among other practices. The authors cited a previous study that found secondary calibrations were inconsistent and unreliable on a protein data set analyzed with a strict molecular clock and a single secondary calibration without associated uncertainty in the age estimate [30]. Morrison [31] later identified the problem of secondary calibrations not being precise enough, resulting in overly wide uncertainty in age estimates. Consequently, applying secondary calibrations might lead to a loss of precision at best, or confounded error at worst, rendering absolute age estimates meaningless.

The Shaul and Graur [30] and Graur and Martin [32] studies predate the common application of the Bayesian approaches implemented in the program Beast [33]. Among the methodological and theoretical advantages that Beast offers, accounting for uncertainty in the fossil calibration by constructing a prior distribution is especially important [5]. This feature is commonly taken advantage of in divergence time analyses that apply a secondary calibration, in which the 95% credible interval (CI) of divergence times from a primary study is used to build a prior distribution in a secondary study (e.g., [28,34–40]). In those studies, normal or uniform prior distributions based on the primary CI are commonly placed on the root node of the secondary study. Such an approach makes the assumption, either explicitly or implicitly, that the uncertainty in age estimates from the primary study will appropriately transfer to the secondary study [34,35,37]. At face value, this approach might escape from the problem of applying an errorless secondary calibration by taking into account the uncertainty in the primary estimate, while allowing for age estimates in groups that lack fossil data.

Applying secondary calibrations has been said to increase the accuracy of the age estimates across a secondary study as long as the estimate was derived from a robust primary calibration [41]. Shaul and Graur's [30] evidence of inconsistency makes intuitive sense, but their methodology has been criticized [31,41]. Furthermore, the consequence of applying a secondary calibration has not been empirically tested with relaxed-clock methods, especially in the context of applying the uncertainty of the primary estimate to a prior distribution in a secondary study. If applying secondary calibrations increases accuracy and allows for the more widespread application of calibrations across systems that lack fossil data, perhaps this practice should be more widely applied. If, on the other hand, the uncertainty of the secondary study deviates greatly from the primary study, perhaps secondary calibrations should not be applied in empirical studies as currently practiced.

The uncertainty from a primary study could transfer to the secondary study in different ways. The uncertainty, as measured by the 95% CIs of the secondary study, might become wider than the primary study, thus indicating even greater uncertainty in age estimates and making the secondary estimates more conservative. The uncertainty might alternatively narrow, and therefore, the secondary study might have estimates that do not include as much uncertainty as the primary estimates, giving a false impression of precision. Alternatively, the secondary study might have similar estimates of uncertainty as the primary study, but their distributions might shift to be younger or older. Any combination of the above outcomes might also be determined (e.g., a wider distribution that shifts to younger ages). Finally, the level of uncertainty in the secondary study could be consistent with that of the primary study in width and position. Only the first and last outcomes are consistent with the assumptions of applying a distribution of age estimates to a secondary study as currently practiced. Given that secondary calibrations are increasingly being applied [42] and the more recent advances in relaxed-clock methods, it is timely to address the consequences of applying secondary calibrations in a relaxed-clock framework.

## Materials and Methods

The consequences of incorporating secondary calibrations in divergence time estimates were assessed with simulated data. The experimental approach was designed to explore the consequences of applying secondary calibrations as is currently practiced in the literature, and not to optimize the best practice of applying secondary calibrations. The simulation approach allowed for assessment of age estimates on data evolved with a known phylogeny under a simple evolutionary process with known ages. A 1500-tip phylogeny was simulated with a pure-birth model [43] that had a birth-rate of 0.7 in the Geiger v1.99–2 [44] and Ape v3.0–8 [45] packages in R [46], and scaled to 70 Ma to be consistent with many family or superfamily level age estimates and species numbers (e.g., Muroidea [47]). The pure-birth tree was then imported into Mesquite v2.75 [48], and used to simulate a 2000 bp DNA matrix based on the HKY [49] DNA substitution model with randomly selected base pair frequencies that were consistent with empirical studies (kappa = 2; base-pair frequencies = 0.30, 0.26, 0.23, 0.21). The data matrix, therefore, consisted of a relatively simple underlying sequence evolution model, and as such, the expected results should be less influenced by complex molecular evolutionary scenarios and phylogenetic uncertainty. Although the ratio of the number of sites to tips might appear low, simulated data is much more information rich compared to empirical data. A maximum likelihood search in PAUP* [50] that applied the HKY model to the simulated DNA data demonstrated the appropriate amount of phylogenetic signal by recovering an identical topology and proportionately similar branch lengths as the simulation tree, plus two additional trees that varied slightly (Robinson-Foulds distances of 4 and 8).

The 2000 bp, 1500-tip DNA sequence data matrix was imported into Beast v1.8.2 [33] along with the pure-birth tree, and divergence times were estimated using the UCLD relax-clock method. I fixed the topology during the initial and all subsequent Beast analyses to simplify the confounding effect that alternative phylogenetic relationships could have on age estimates and because the large number of tips made reaching stationarity difficult in a reasonable timeframe. Analyses were ran to obtain effective sample sizes greater than 300, although this was not reached for the UCLD standard deviation or coefficient of variation. I applied a custom R script to randomly sample 29 nodes plus the root node from the original pure-birth tree to calibrate the 1500-tip tree, which resulted in an even distribution of calibration points across the tree in both deep and shallow nodes. This even distribution allowed for an increased chance that the subtrees were located near a calibration point on the primary tree and it also decreased the interval between calibration points, which would have increased credible intervals otherwise [22]. I applied lognormal priors for all calibrations with a standard deviation of one. A Yule speciation prior and the HKY DNA-substitution-model were assigned, and I ran the UCLN model for 100 million generations, sampling from the posterior distribution every 2000 generations. The first half of the posterior distribution was discarded in TreeAnnotator [33] to allow the program enough memory to complete its summary of the posterior distribution. The resulting maximum clade credibility (MCC) tree, which I will refer to as the primary tree, was then used in all comparisons (trees, DNA data, and custom R scripts can be downloaded from GSU Digital Commons: http://digitalcommons.georgiasouthern.edu/biology-data/1/).

The secondary trees were compared to the primary tree and not the original pure-birth tree for several reasons. The main question of this study was whether the amount of uncertainty in posterior distributions, as applied as prior distributions, are appropriately transferred in secondary calibration studies. As such, as posterior distribution of times were needed and the pure-birth tree provided only point estimates. Although comparisons could have been made with the primary and secondary estimates to the pure-birth tree, such a comparison adds an element of unnecessary complexity and the deviation between the Beast estimates of the primary and secondary trees from the pure-birth tree is expected to be similar.

From the primary tree, I applied a second custom script in R that depended on the Phytools package v0.3–93 [51] to randomly extract 100 clades without replacement on the condition that they contained at least 20 tips. Subsampling clades with 20 or more tips mimics the sampling of studies that apply secondary calibrations and allowed for the study to avoid overestimation of rates associated with calibrating young nodes [29]. The 100 subsampled trees contained all members of their respective clade, and I therefore do not expect incomplete sampling bias to influence age estimates. The secondary trees were individually imported into Beast along with their corresponding DNA data matrix from the DNA simulation, once again simplifying the comparisons by not confounding error because of different gene histories or evolutionary rates.

Published studies have applied both uniform and normal distributions as secondary priors, despite the fact that these distributions might perform poorly [22]. A uniform prior distribution was applied to the secondary-tree root-node from the primary tree's minimum and maximum 95% CI values. Because the shape of the prior distribution can influence age estimates, I also explored the impact of applying normal distribution priors in the secondary study by conducting duplicated analyses of every 10^th^ random replicate with a normal distribution prior in which 95% of the prior distribution of the secondary study spanned the 95% CI of the primary tree. Beast analyses were conducted with a Yule speciation prior, the HKY DNA-substitution-model, and analyses were ran with the UCLN model for 30 million generations for replicates that included less than 200 tips, and 60 million generations for data sets comprised of 200 tips or greater. The posterior distribution was sampled every 3000 generations in all analyses. Analyses were conducted on local machines and on the Cipres Science Gateway [52]. The first half of the posterior distribution was discarded, as it was in the primary tree, and the second half was summarized in TreeAnnotator.

Applying multiple calibrations will likely lead to more precise age estimates [32], given the calibrations are not in conflict with one another [22]. I tested whether more consistent age estimates could be estimated if an additional secondary calibration was applied. A second node was calibrated on every 10^th^ replicate, in which I calibrated the node that was two nodes tipward from the root node on the right side. The average distance between the two calibrated nodes was 8.920 million years. This sampling strategy mimics those studies that would apply multiple secondary calibrations, which would be more likely to calibrate deeper versus shallower nodes. Uniform prior distributions were applied for both nodes, and the Beast analyses were conduct as above.

Additional analyses were conducted to determine whether the primary results held if data were simulated under a relaxed-clock model. The primary pure-birth tree was imported into the R package NELSI [53] and branch lengths were rescaled according to the UCLD model with a lognormal mean rate of 0.020 and standard deviation of 2.017. The rescaled tree was used to simulate a 3000-bp DNA character matrix, which was evolved under the HKY model as above. Differences between the full tree and subsampled trees were compared for every 10^th^ replicate as above.

If the assumption that taking into account the uncertainty of a primary study transfers appropriate uncertainty to the secondary study is supported, I predict that (1) CIs will be nearly the same width or wider in the secondary study, (2) CIs will be nearly overlapping, and (3) the median age estimates will be approximate. To measure the difference in estimates, I summed the absolute values of the difference in the minimum 95% highest posterior density (HPD) interval estimate across all nodes, as well as the maximum values, the median values, and the total width of the 95% CI per secondary tree replicate by writing a third custom script in R that took advantage of the Phyloch package v1.5 (unpublished package, C. Heibl). The difference between median estimates was assessed with a Student's paired T-test in R with Bonferroni corrections for multiple comparisons. The differences in CI widths between primary and secondary estimates was assessed by pooling together all estimates from the 100 replicated searches with a uniform prior distribution and subjecting these estimates to a Student's paired T-test. I also plotted the raw values of these differences as a function of median clade ages to determine the effects of variation in age estimates from the root to the tips of the tree. I investigated the effect that root ages and the number of species has on estimates by conducting linear regressions in which independent analyses included the dependent variables of minimum and maximum CI ages, median ages, and CI widths were regressed on the secondary tree root age and the number of species. Bonferroni corrections were applied to account for multiple comparisons. The assumptions of the frequentist statistical approaches applied here are likely to be violated due to non-independence of overlapping nodes and should be carefully interpreted; however, they are applied here to describe the broad patterns in the data.

## Results

**Fig 1:**
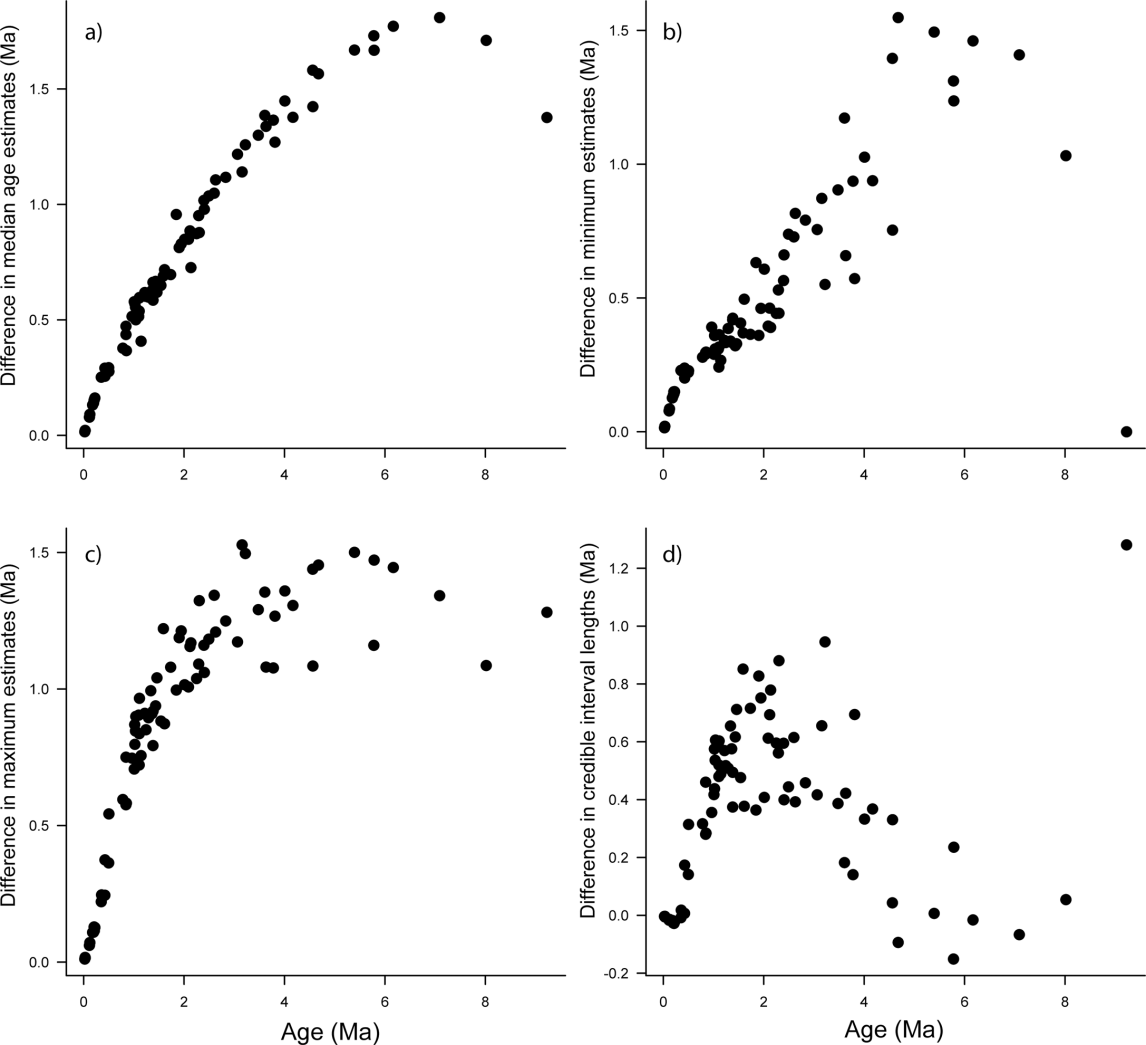
Randomly chosen example of ages estimated with a secondary calibration compared to those from the primary tree in one replicate (node 2509 on primary tree). All graphs were constructed by comparing the secondary estimates to the primary estimates so that positive values on the y-axis for A–C indicate younger age estimates in the secondary study, and positive values on the y-axis for D indicate shorter credible interval (CI) in the secondary tree. Y-axes indicate (A) Difference in median age estimates. (B) Difference in the minimum age estimates based on the 95% CI. (C) Difference in the maximum age estimates based on the 95% CI. (D) Difference in CI widths of primary versus secondary study.

**Fig 2:**
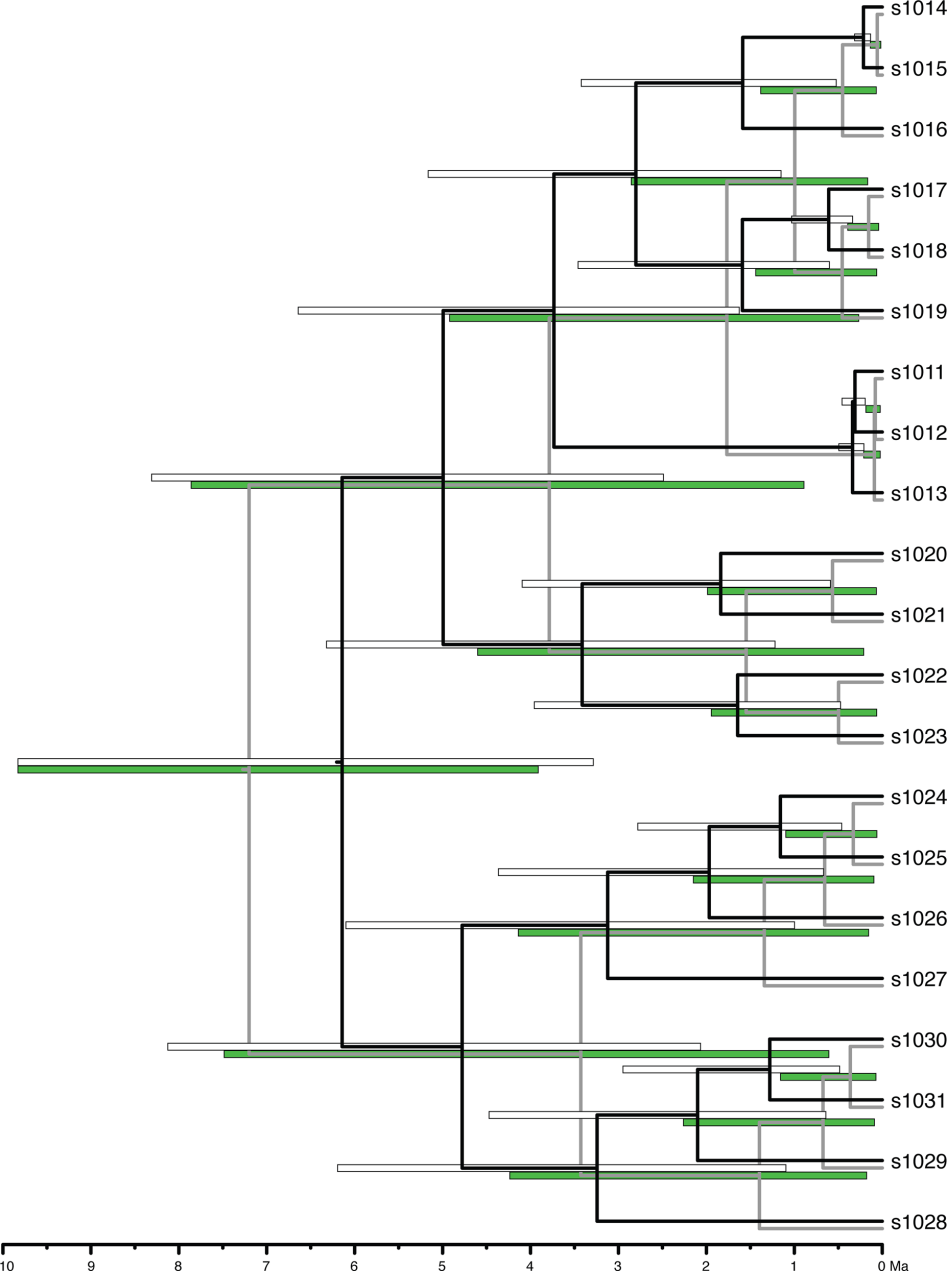
Chronogram of one example replicated subtree (based on node 2518) with credible intervals of the primary estimates (white error bars) and secondary estimates (green error bars). The 95% highest posterior density (HPD) tree is represented in black for the primary estimate, and grey in the secondary estimate. Overall, secondary estimates were found to be younger than primary estimates, and the CIs were shorter. Some nodes, such as those associated s1011, s1012, and s1013, exhibit CIs between the primary and secondary analyses that do not overlap. In this example, the 95% HPD estimate for the root node is older in the secondary analysis than in the primary, although older ages for root nodes are not always inferred.

**Fig 3:**
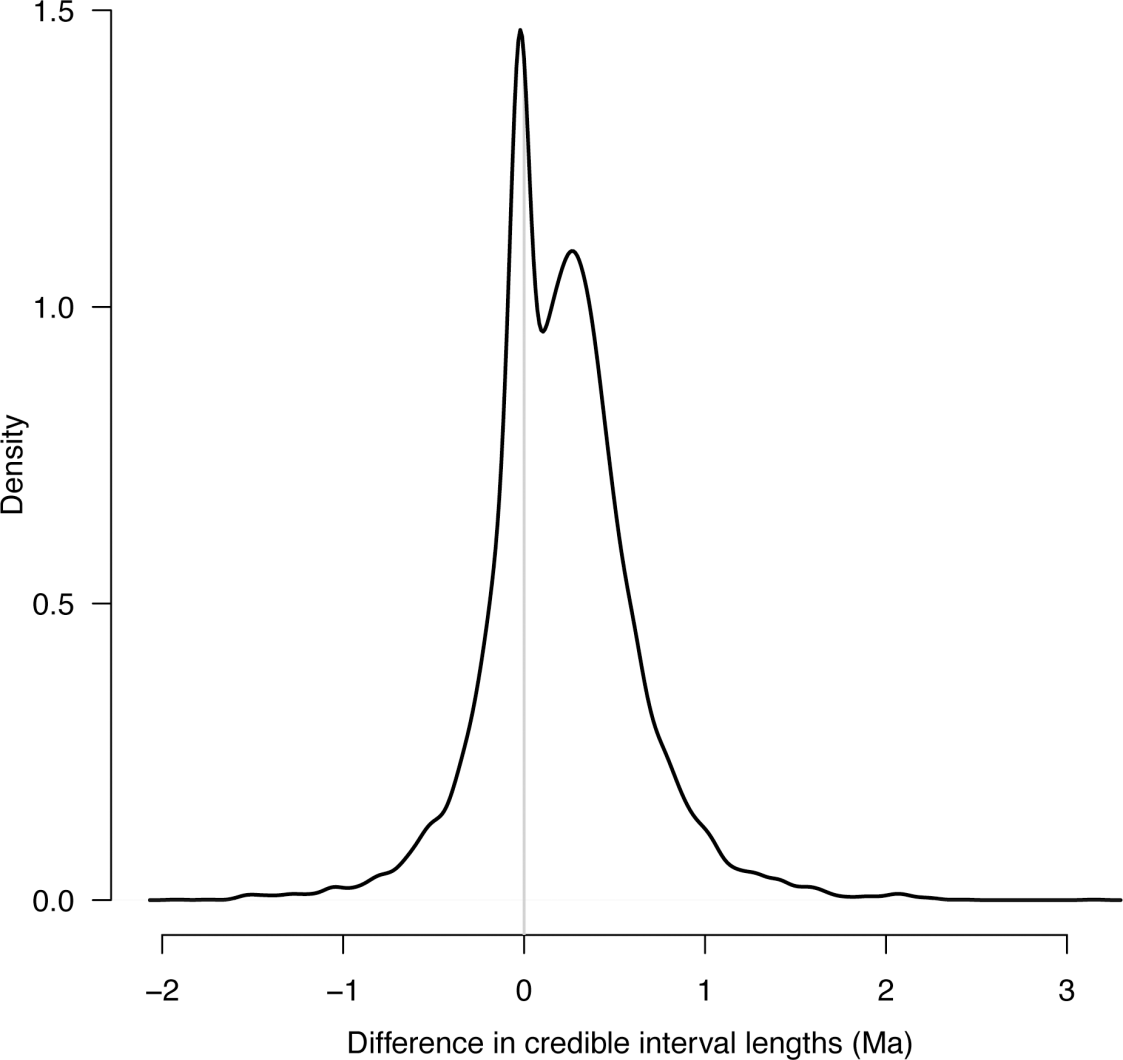
Density plot displaying the differences in credible interval estimates of nodes from the primary and secondary analyses. If uncertainty from the primary study transferred to the secondary study, credible intervals for each comparison should approximately equal zero (represented by grey line); however, the majority of estimates fall above, indicating that the primary tree has wider credible intervals on average than the secondary study.

**Fig 4:**
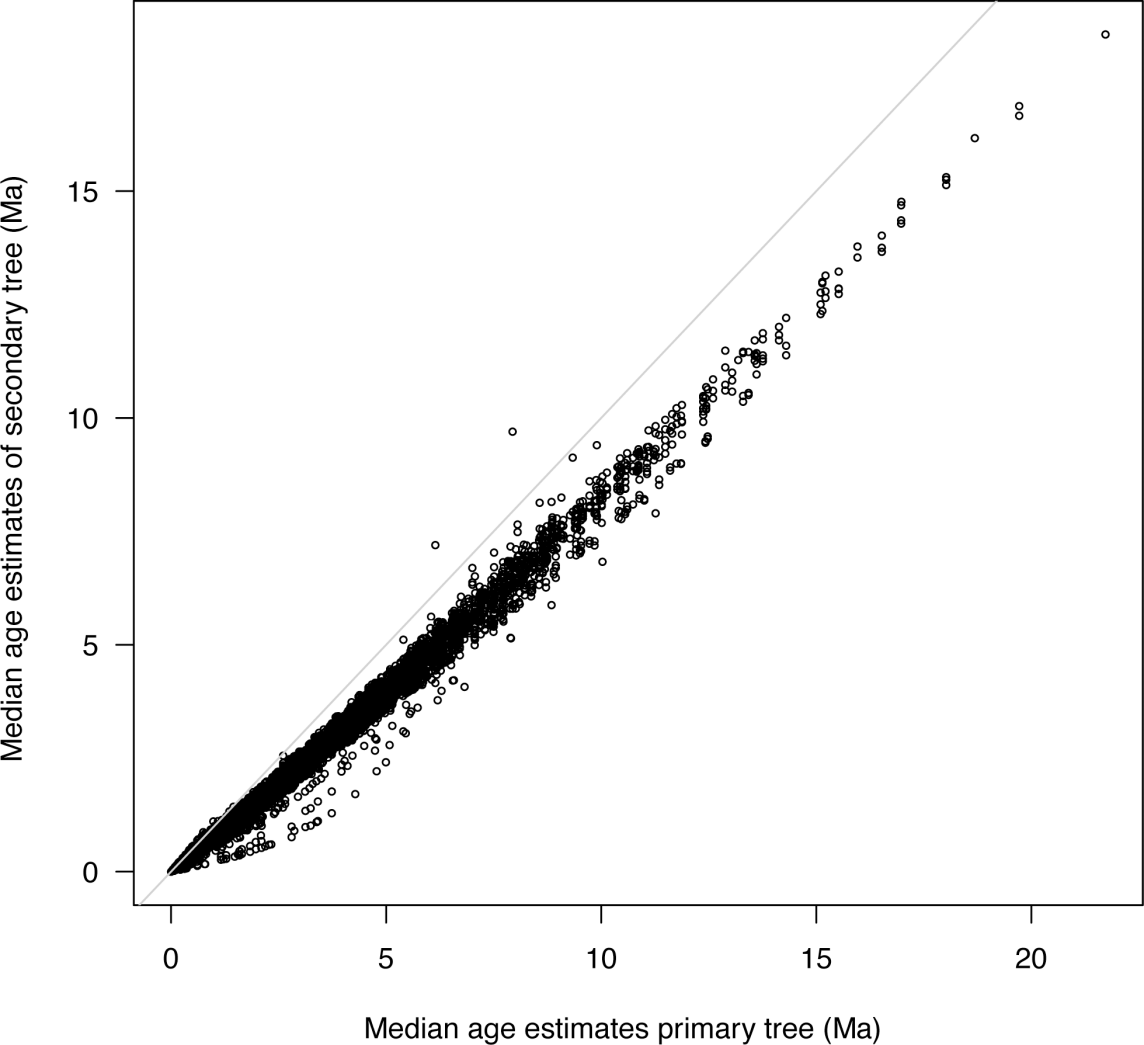
Median age estimates of nodes from the primary and secondary analyses. If age estimates were approximate, age estimates would fall near the grey line (slope = 1, intercept = 0); however, node ages are generally younger in the secondary than in the primary analysis.

**Fig 5:**
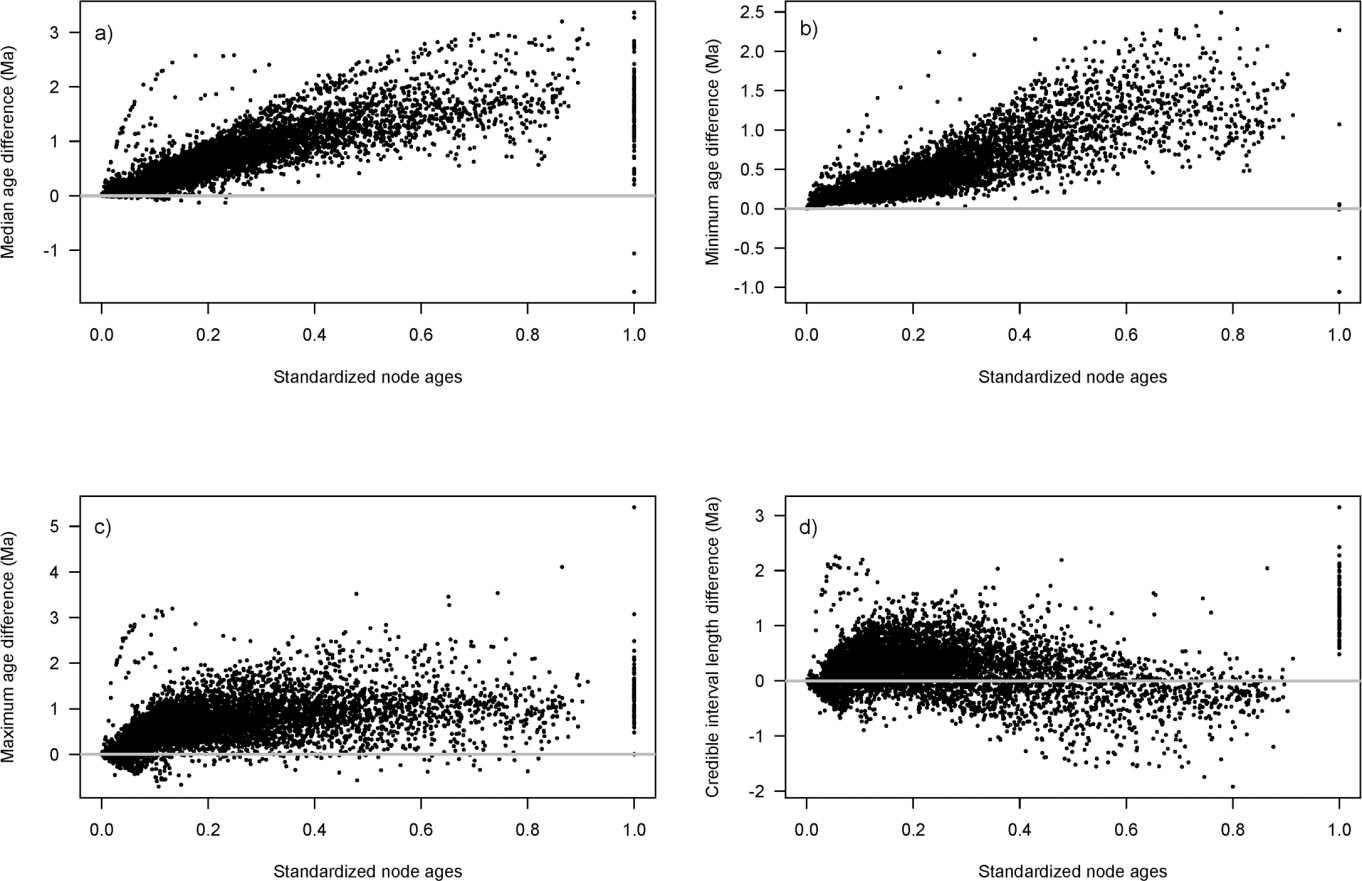
Differences in age estimates compared to standardized node ages in all 100 replicates based on the uniform distribution. Node ages of all replicates have been standardize so that the root node = 1 and the tips = 0 (so comparisons can be made across different replicates of different root ages). (A) Difference in median age estimates, (B) difference in minimum age estimates, (C) difference in maximum age estimates, and (D) difference in the credible interval widths. The null hypothesis of no difference in age estimates is indicated with a grey line on all plots.

**Fig 6:**
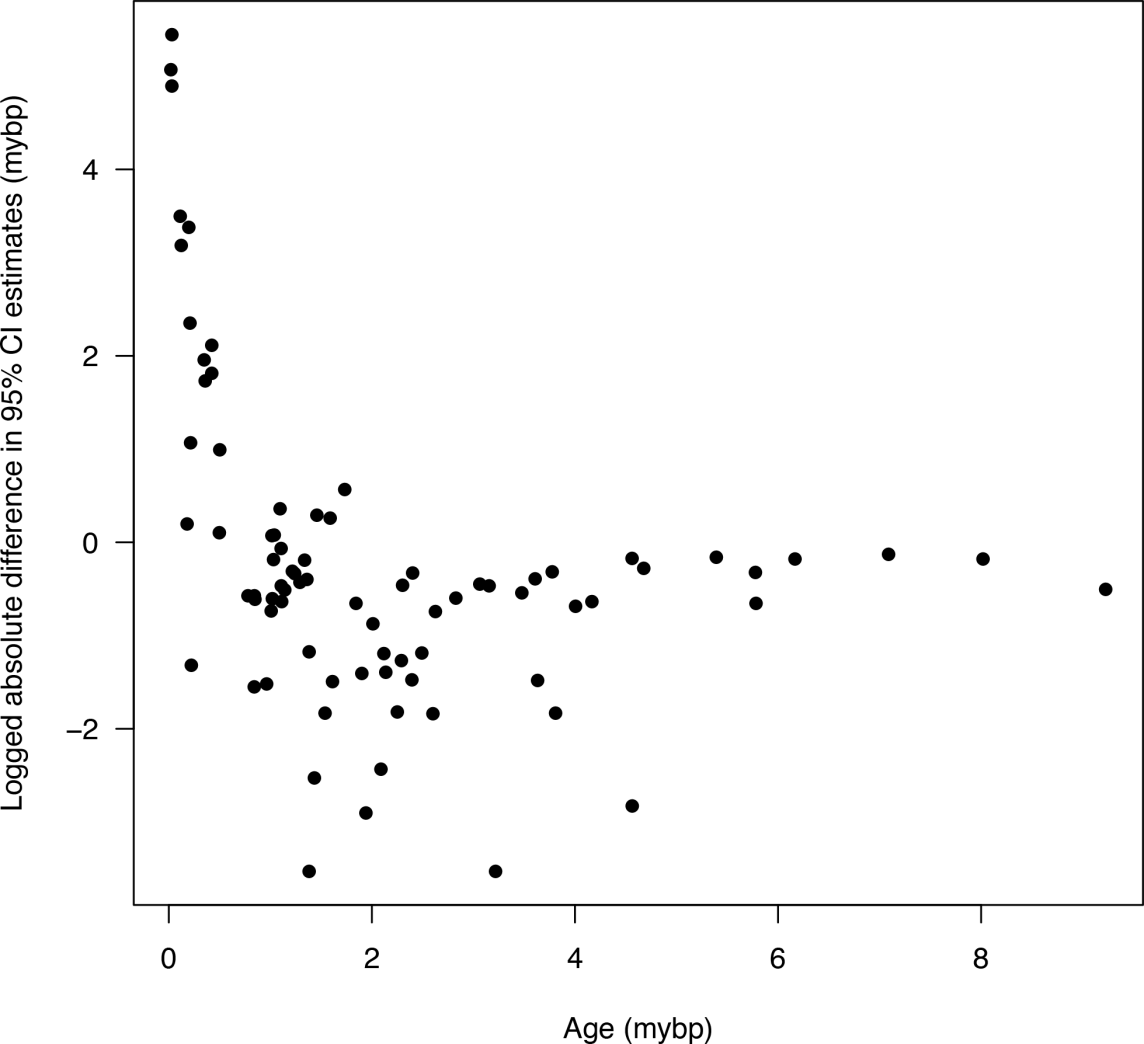
Difference in credible intervals after being standardized by the median node age for a representative replicate. Plot represents node 2509, as in Fig 1, and should be compared to Fig 1D.

Age estimates of secondary trees, as measured with the 95% HPD median ages and CI values, were neither identical to the primary tree, nor were the secondary trees' CIs wider (Figs 1–5). The differences between median age estimates were significantly younger on average in all replications based on the T-tests (*P* < 0.05; Fig 4), and all but three replicates remained significant at the alpha = 0.05 level when the conservative Bonferroni corrections were applied to correct for multiple comparisons. Greater magnitude of variation in age estimates were associated with estimates closer to the root of the tree (Fig 5), however, the opposite pattern was observed when values were standardized by the median node ages (Fig 6). Increased variance of age estimates was determined for nodes closer to the root, in which the age estimates were mostly younger for the secondary estimates measured by the minimum, maximum, and median values from the 95% CI (Fig 5A–5C). Standardized age estimates for the CI widths showed a sinusoidal relationship, in which comparisons at the tips of the phylogeny had little difference, CIs then became shorter, peaking around 0.2 standardized node age, followed generally by secondary age estimates becoming longer (peaking around 0.7 standardized node age), and then becoming shorter again near the root (Fig 5D). A T-test determined that there were significantly different CI width estimates between the primary and secondary estimates (t = –46.50, df = 8871, *P* < 0.01).

**Fig 7:**
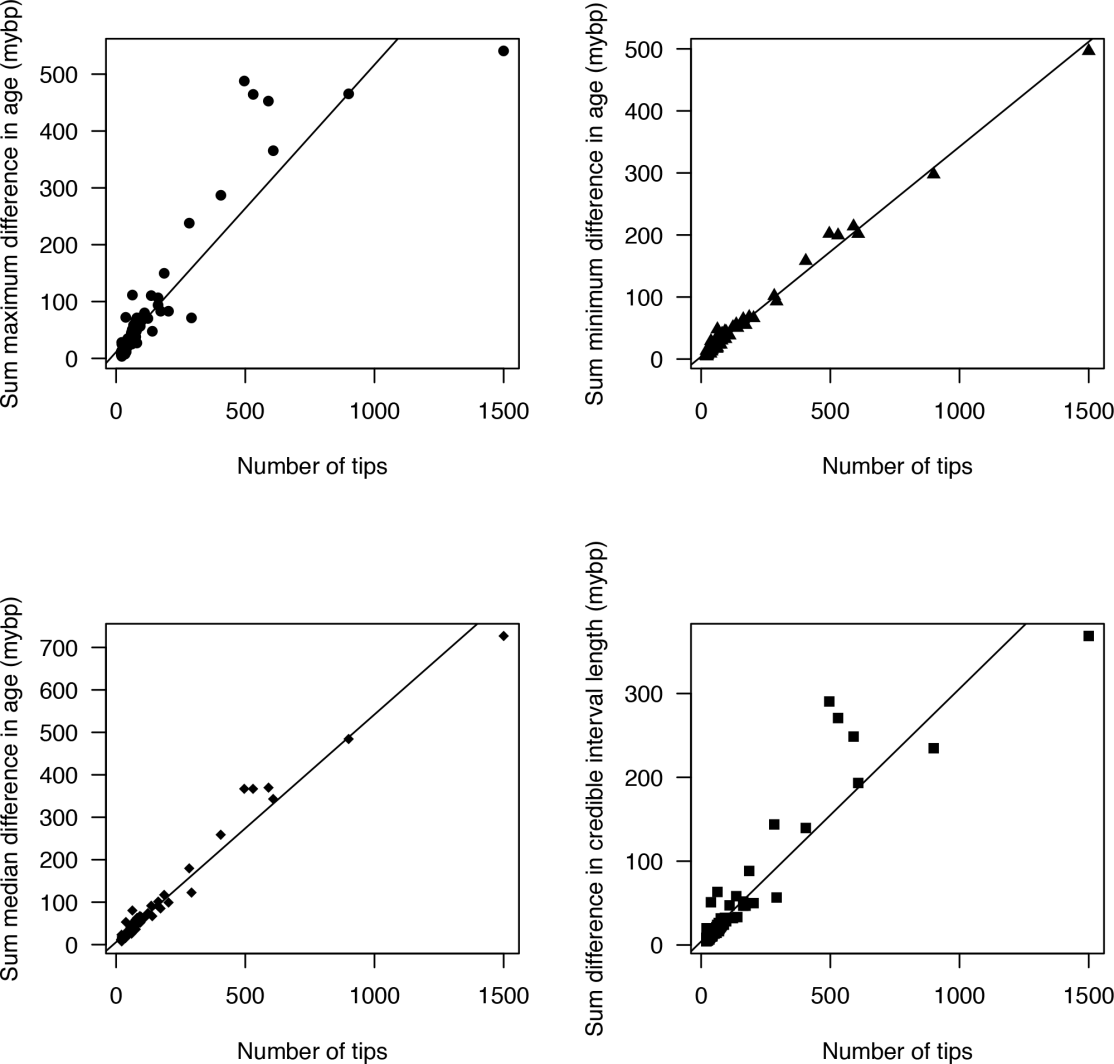
Relationship in magnitude between the number of tips and (A) maximum difference in age estimates, (B) minimum difference in age estimates, (C) median difference in age estimates, and (D) difference in credible interval widths. A linear model fit onto all data sets revealed a significant positive relationship after Bonferroni corrections (*P* < 0.05).

Both clade age and number of tips were positively correlated with the summed difference in median age estimates (*P* < 0.01). Older clades with more tips were associated with the greatest amount of error (Fig 7). The difference in error was not identical for the minimum as they were for the maximum estimates. The minimum estimates and number of tips revealed a tight linear relationship (as did the median estimates), whereas the maximum estimates and the number of tips revealed greater variation among age estimates (as judged by residuals), especially as the number of tips increased (Fig 7). The summed difference in CI widths among replicates also revealed greater variation in estimates as the number of tips increased.

Greater error in age estimates were inferred in analyses that applied a normal prior distribution in secondary studies compared to results based on a uniform prior on average (Table 1). The absolute summed differences between primary and secondary analyses were less on average when a uniform prior was applied than when a normal prior was applied. All analyses, regardless of the prior distribution shape, had significantly different median estimates.

**Table 1:** Comparisons of every 10^th^ replicate showing differences in the prior distribution, number of calibrated nodes, and relaxed clock simulations. Average (Avg.) minimum (Min), maximum (Max), median, and credible interval (CI) values are the averaged absolute summed differences between primary and secondary estimates in time units (Ma). The uniform and normal prior distribution replicates and relaxed clock simulations applied a single calibration, whereas the two node replicates applied two calibrations per replicate, both of which have a uniform prior distribution. The T-test results are the proportion of replicates that resulted in a significant value at *P* < 0.05.

	Uniform	Normal	2 nodes (uniform)	Relaxed
Avg. Min	51.72	98.99	43.48	52.40
Avg. Max	78.66	80.29	85.21	84.54
Avg. Median	79.85	87.03	73.60	80.30
Avg. CI	45.53	55.39	47.07	55.29
T-test median	100%	100%	100%	100%

Analyses that incorporated two secondary calibrated nodes were associated with less absolute summed differences of minimum and median age estimates on average than estimates based on a single node and analyses based on a normal distribution (Table 1). Greater differences in analyses that applied two calibrated nodes were associated with maximum age estimates. Credible intervals were associated with greater difference in analyses that applied two secondary calibrated nodes than those that applied a single node, but not as great as analyses that applied a normal distribution.

To test the robustness of the primary results to rate heterogeneity, data were simulated under the UCLN relaxed-clock model and reanalyzed. Comparisons made between the primary and secondary trees for these data again identified significant differences between primary and secondary estimates in all replicates (Table 1), and difference measures were on average greater in the relaxed-clock data than uniform-prior estimates based on simulated data with correlated and constant divergence times (Table 1).

## Discussion

Divergence time estimates have had a profound influence on biological inference. These approaches have tied evolutionary events to geologic and climate events, have allowed us to model expected change over time to study character evolution and lineage diversification, and they are the basis of most comparative studies [2,3,42]. It is therefore understandable why researchers have applied alternative calibration strategies when their study system lacks fossil data. Yet the question remains, what is the cost of implementing secondary calibrations in empirical studies? If our aim is to test hypotheses with robust methods that produce reliable age estimates, the cost of applying secondary calibrations might be too high with our current methods.

I proposed in the introduction that only results with approximately similar age estimates or wider CIs would be consistent with the current practice of applying secondary calibrations. I determined neither to be true. The CIs, which most studies apply to represent uncertainty in age estimates, are on average significantly narrower in the secondary study (Fig 3), giving the false impression of precision, and they are generally shifted to younger ages (Fig 4). Even with Bonferroni corrections for multiple comparisons, 97% of the secondary analyses were inferred to have significantly different age estimates, a proportion much too high to consider reasonable. Clearly, the assumption that applying secondary calibrations will account for the uncertainty from the primary study is invalid.

The result that variation in age estimates, as judged by the 95% CI, is lost when secondary calibrations are applied is anything but surprising. Applying a distribution that consists of only 95% of the credible interval, as is commonly practiced, will by definition remove 5% of the variation. Variation in age estimates is also not consistent across all nodes. Tipward nodes were associated with less variation when considering magnitude, but were associated with much greater variation when standardized by the median node age (Fig 6). The higher variation in tipward clades might be due to more uncertain rate estimates associated with less information at shallow nodes. The greater variation in magnitude associated with deeper nodes (Figs 1 and 5), as well as the greater relative variation in tipward nodes makes predictions of how secondary calibrations will misestimate ages more tenuous. For each node, how the CIs vary was also inconsistent, in which applying secondary calibrations had a much greater effect on maximum compared to minimum age estimates (Figs 1 and 5).

I primarily explored the effects of applying a uniform prior because it is common practice in the literature and is more conservative in the comparisons made in this study, but I also explored the effects of applying a normal prior. Greater error was associated with normal than uniform distributions (Table 1), despite its' common use in secondary calibration studies [28]. Using the 95% CI from the primary study to construct a prior distribution in a secondary study automatically reduces the total uncertainty assigned to the prior, and applying a normal distribution can reduce the uncertainty even greater by assigning lower prior probabilities near the tails. The choice of what distribution to model for a secondary calibration is often overlooked. Applying a prior distribution that is most similar to the posterior distribution (e.g., lognormal [28]) is preferable, but seldom done, and more work is needed to understand the interactions among the prior distributions of secondary calibrations and rate priors.

This study was designed to simplify confounding factors that can increase discordance among age estimates when applying secondary calibrations. The true species tree was applied in all analyses and the topology was fixed while ages were estimated, thus absolving phylogenetic uncertainty in topology. Primary and secondary trees were estimated with the same DNA matrix with a known and simple substitution model that resolved lineages at both deep and shallow positions [24,26]. The secondary analyses were also conducted with all species from their respective clade [27], calibrations were taken from a tree with a constant substitution rate across all lineages, and the primary ages were estimated with numerous calibrations of known, precise ages that were located across the phylogeny [4,14–16,32] and correctly placed [4]. The results presented here, therefore, might be the best chance to obtain consistent ages between primary and secondary estimates, and results from empirical data sets could be associated with even greater error due to the above effects. Analyses that applied more-complex data simulated with a relaxed-clock model, for example, were associated with greater error than those simulated with a constant substitution rate (Table 1).

A single secondary calibration was applied in this study to mimic common practices in divergence time estimation. It has long been appreciated that age estimates based on a single calibration are more prone to error than one in which multiple, but consistent, calibrations are applied [14,32]. One approach taken to mitigate this error is to apply a combination of primary and secondary calibrations. Although this may result in narrower error estimates around age estimates [41], as shown here, this may be due to incorrect inferences of error rather than more precise estimates, or due to conflicting age estimates [22]. Following the logic of Graur and Martin [32] that applying only a single calibration point leads to greater error, one would expect that applying a second secondary calibration would generate age estimates that are more similar to those from the primary analysis as long as they were consistent. This expectation was not met. The primary and secondary estimates were more similar, but only for the minimum and median estimates (Table 1). The maximum age estimates and CIs were actually more divergent when a second calibration was applied. This result suggests that multiple secondary calibrations cannot rescue secondary age estimates.

An alternative approach to secondary calibrations is to sample more inclusively until a data set includes the focal group as well as a more distantly related clades that includes fossil data. This approach has the advantage of estimating divergence times in groups without fossil data while directly applying primary fossil ages. Data sets now exist that facilitate applying a wider set of fossil data, such as the Paleobiology database (http://paleobiodb.org/), Fossilworks (http://fossilworks.org), Palaeontologia Electronica (http://palaeo-electronica.org), Parham et al. [54], Weir and Schluter [29], and Magallón and Castillo [55]. This approach, however, might have some undesired drawbacks. As larger clades are sampled (often from GenBank accessions), the probability of sampling clades with missing species increases, which might affect divergence time estimates [27]. These larger data sets, which will often require a multigene approach that includes fast and slow evolving genes to resolve deep and shallow nodes, also runs the risk of containing missing data for particular genes, which might be problematic in branch length estimates [56], as well as saturation in deeper relationships of fast evolving genes [26]. This approach will also contain increased uncertainty in age estimates as patristic distance from the calibration point increases [22,23,57], which might generate CIs that are too wide to reject null hypotheses (although for the correct reason) [31]. Consequently, although this approach is likely to produce more reliable results that appropriately incorporate uncertainty compared to applying secondary calibrations, important sampling issues need to first be considered.

A closer examination of the error generated by applying a secondary calibration revealed that it is not completely confounded or intractable. The difference between primary and secondary estimates behaved consistently across replicates and is dependent on the number of tips or the root age. The effects of applying a secondary calibration could be parameterized in divergence time estimates. For example, the distributions in Figs 3 and 5 could be used to construct a secondary calibration distribution to take into account the additional uncertainty in age estimates across nodes. Noting that the impact of secondary calibrations changes across nodes, the consequences of secondary calibrations could be accounted for, which would allow for study systems that do not have fossil data to generate age estimates that reasonably account for the uncertainty associated with secondary calibrations. Thus, the problem might not be using secondary calibrations, but rather, using them without taking into account the additional uncertainty. Until such methods are developed, applying secondary calibrations in studies that test hypotheses that include a time component should be discouraged.

In conclusion, secondary calibrations fail to accurately account for the variation and uncertainty that was inferred in the primary study. The prior distribution that is accounted for in the secondary analysis is not identical to the error estimates in the primary study, and this disconnection generates biased age estimates. Incorrect age and error estimates are likely to be exasperated as researchers apply truncated and different prior distributions than the posterior distributions that were estimated in the primary study. Empirical studies are likely to include much more complex data than what were analyzed here, such as rate heterogeneity among lineages, deep tree distances from calibrated root node to tips, and secondary calibrations placed on non-root nodes, producing the potential to estimate secondary age estimates that vary greatly from primary estimates. As such, age estimates from secondary calibrations should not be trusted until methodological advances are developed to account for uncertainty in these estimates.

## References

[1] Zuckerkandl E, Pauling L (1965) Evolutionary divergence and convergence in proteins In: Bryson V, Vogel HJ, editors. Evolving Genes and Proteins. New York: Academic Press pp. 97–166.

[2] Bromham L, Penny D (2003) The modern molecular clock. Nature Reviews Genetics 4: 216–224. 12610526 10.1038/nrg1020

[3] Welch JJ, Bromham L (2005) Molecular dating when rates vary. Trends in Ecology & Evolution 20: 320–327. 16701388 10.1016/j.tree.2005.02.007

[4] Magallón S (2004) Dating lineages: Molecular and paleontological approaches to the temporal framework of clades. International Journal of Plant Sciences 165: S7–S24.

[5] Ho SYW, Phillips MJ (2009) Accounting for calibration uncertainty in phylogenetic estimation of evolutionary divergence times. Systematic Biology 58: 367–380. 10.1093/sysbio/syp035 20525591

[6] Yoder AD, Yang Z (2000) Estimation of primate speciation dates using local molecular clocks. Molecular Biology and Evolution 17: 1081–1090. 10889221 10.1093/oxfordjournals.molbev.a026389

[7] Sanderson MJ (2002) Estimating absolute rates of molecular evolution and divergence times: A penalized likelihood approach. Molecular Biology and Evolution 19: 101–109. 11752195 10.1093/oxfordjournals.molbev.a003974

[8] Sanderson MJ (2003) r8s: Inferring absolute rates of molecular evolution and divergence times in the absence of a molecular clock. Bioinformatics 19: 301–302. 12538260 10.1093/bioinformatics/19.2.301

[9] Thorne JL, Kishino H, Painter IS (1998) Estimating the rate of evolution of the rate of molecular evolution. Molecular Biology and Evolution 15: 1647–1657. 9866200 10.1093/oxfordjournals.molbev.a025892

[10] Kishino H, Thorne JL, Bruno WJ (2001) Performance of a divergence time estimation method under a probabilistic model of rate evolution. Molecular Biology and Evolution 18: 352–361. 11230536 10.1093/oxfordjournals.molbev.a003811

[11] Thorne JL, Kishino H (2002) Divergence time and evolutionary rate estimation with multilocus data. Systematic Biology 51: 689–702. 12396584 10.1080/10635150290102456

[12] Drummond S, Ho S, Phillips M, Rambaut A (2006) Relaxed phylogenetics and dating with confidence. PLOS Biology 4: e88 16683862 10.1371/journal.pbio.0040088 PMC1395354

[13] Lemey P, Rambaut A, Drummond AJ, Suchard MA (2009) Bayesian phylogeography finds its roots. PLoS Computational Biology 5: e1000520 10.1371/journal.pcbi.1000520 19779555 PMC2740835

[14] Sauquet H, Ho SYW, Gandolfo MA, Jordan GJ, Wilf P, Cantrill DJ, et al (2012) Testing the impact of calibration on molecular divergence times using a fossil-rich group: The case of *Nothofagus* (Fagales). Systematic Biology 61: 289–313. 10.1093/sysbio/syr116 22201158

[15] Yang Z, Yoder AD (2003) Comparison of likelihood and Bayesian methods for estimating divergence times using multiple gene loci and calibration points, with application to a radiation of cute-looking mouse lemurs. Systematic Biology 52: 705–716. 14530137 10.1080/10635150390235557

[16] Rannala B, Yang Z (2007) Inferring speciation times under an episodic molecular clock. Systematic Biology 56: 453–466. 17558967 10.1080/10635150701420643

[17] Magallón S (2010) Using fossils to break long branches in molecular dating: A comparison of relaxed clocks applied to the origin of angiosperms. Systematic Biology 59: 384–399. 10.1093/sysbio/syq027 20538759

[18] Rutschmann F, Eriksson T, Salim KA, Conti E (2007) Assessing calibration uncertainty in molecular dating: The assignment of fossils to alternative calibration points. Systematic Biology 56: 591–608. 17654364 10.1080/10635150701491156

[19] Duchêne S, Lanfear R, Ho SYW (2014) The impact of calibration and clock-model choice on molecular estimates of divergence times. Molecular Phylogenetics and Evolution 78: 277–289. 10.1016/j.ympev.2014.05.032 24910154

[20] Near TJ, Sanderson MJ (2004) Assessing the quality of molecular divergence time estimates by fossil calibrations and fossil-based model selection. Philosophical Transactions of the Royal Society B-Biological Sciences 359: 1477–1483. 10.1098/rstb.2004.1523 PMC1693436 15519966

[21] Andujar C, Soria-Carrasco V, Serrano J, Gomez-Zurita J (2014) Congruence test of molecular clock calibration hypotheses based on Bayes factor comparisons. Methods in Ecology and Evolution 5: 226–242.

[22] Reis MD, Yang Z (2013) The unbearable uncertainty of Bayesian divergence time estimation. Journal of Systematics and Evolution 51: 30–43.

[23] Rannala B, Yang Z (2007) Inferring speciation times under an episodic molecular clock. Systematic Biology 56: 453–466. 17558967 10.1080/10635150701420643

[24] Schenk JJ, Hufford L (2010) Effects of substitution models on divergence time estimates: Simulations and an empirical study of model uncertainty using Cornales. Systematic Botany 35: 1–15.

[25] Phillips MJ (2009) Branch-length estimation bias misleads molecular dating for a vertebrate mitochondrial phylogeny. Gene 441: 132–140. 10.1016/j.gene.2008.08.017 18809474

[26] Zheng Y, Peng R, Kuro-o M, Zeng X (2011) Exploring patterns and extent of bias in estimating divergence time from mitochondrial DNA sequence data in a particular lineage: A case study of salamanders (Order Caudata). Molecular Biology and Evolution 28: 2521–2535. 10.1093/molbev/msr072 21422243

[27] Linder PH, Christopher RH, Rutschmann F (2005) Taxon sampling effects in molecular clock dating: An example from the African Restionaceae. Molecular Phylogenetics and Evolution 35: 569–582. 15878126 10.1016/j.ympev.2004.12.006

[28] Ho SYW (2007) Calibrating molecular estimates of substitution rates and divergence times in birds. Journal of Avian Biology 38: 409–414.

[29] Weir JT, Schluter D (2008) Calibrating the avian molecular clock. Molecular Ecology 17: 2321–2328. 10.1111/j.1365-294X.2008.03742.x 18422932

[30] Shaul S, Graur D (2002) Playing chicken (*Gallus gallus*): Methodological inconsistencies of molecular divergence date estimates due to secondary calibration points. Gene 300: 59–61. 12468086 10.1016/s0378-1119(02)00851-x

[31] Morrison DA (2010) Counting chickens before they hatch: Reciprocal consistency of calibration points for estimating divergence dates. ArXiv e-prints: 1001.3586.

[32] Graur D, Martin W (2004) Reading the entrails of chickens: Molecular timescales of evolution and the illusion of precision. Trends in Genetics 20: 80–86. 14746989 10.1016/j.tig.2003.12.003

[33] Drummond AJ, Rambaut A (2007) BEAST: Bayesian evolutionary analysis by sampling trees. BMC Evolutionary Biology 7: 214 17996036 10.1186/1471-2148-7-214 PMC2247476

[34] Kuriyama T, Brandley MC, Katayama A, Mori A, Honda M, Hasegawa M, et al (2011) A time-calibrated phylogenetic approach to assessing the phylogeography, colonization history and phenotypic evolution of snakes in the Japanese Izu Islands. Journal of Biogeography 38: 259–271.

[35] Ryberg M, Matheny PB (2011) Dealing with incomplete taxon sampling and diversification of a large clade of mushroom-forming fungi. Evolution 65: 1862–1878. 10.1111/j.1558-5646.2011.01251.x 21729044

[36] Siler CD, Oaks JR, Welton LJ, Linkem CW, Swab JC, Diesmos AC, et al (2012) Did geckos ride the Palawan raft to the Philippines? Journal of Biogeography 39: 1217–1234.

[37] Kornilios P, Giokas S, Lymberakis P, Sindaco R (2013) Phylogenetic position, origin and biogeography of Palearctic and Socotran blind-snakes (Serpentes: Typhlopidae). Molecular Phylogenetics and Evolution 68: 35–41. 10.1016/j.ympev.2013.03.009 23523862

[38] Ruiz-Sanchez E, Specht CD (2013) Influence of the geological history of the Trans-Mexican Volcanic Belt on the diversification of *Nolina parviflora* (Asparagaceae: Nolinoideae). Journal of Biogeography 40: 1336–1347.

[39] Zhao Y, Qi Z, Ma W, Dai Q, Li P, Cameron KM, et al (2013) Comparative phylogeography of the *Smilax hispida* group (Smilacaceae) in eastern Asia and North America: Implications for allopatric speciation, causes of diversity disparity, and origins of temperate elements in Mexico. Molecular Phylogenetics and Evolution 68: 300–311. 10.1016/j.ympev.2013.03.025 23578597

[40] Schenk JJ (2013) Biogeographical diversification of *Mentzelia* section *Bartonia* in western North America. Journal of Biogeography 40: 455–465.

[41] Hedges SB, Kumar S (2004) Precision of molecular time estimates. Trends in Ecology & Evolution 20: 242–247. 10.1016/j.tig.2004.03.004 15109778

[42] Hipsley CA, Müller J (2014) Beyond fossil calibrations: Realities of molecular clock practices in evolutionary biology. Frontiers in Genetics 5: 1–11. 24904638 10.3389/fgene.2014.00138 PMC4033271

[43] Yule GU (1924) A mathematical theory of evolution, based on the conclusions of Dr. J. C. Willis. Philosophical Transactions of the Royal Society B 213: 21–87.

[44] Harmon L, Weir J, Brock C, Glor R, Challenger W (2008) GEIGER: Investigating evolutionary radiations. Bioinformatics 24: 129–131. 18006550 10.1093/bioinformatics/btm538

[45] Paradis E, Claude J, Strimmer K (2004) APE: Analyses of phylogenetics and evolution in R language. Bioinformatics 20: 289–290. 14734327 10.1093/bioinformatics/btg412

[46] R Development Core Team (2005) R: A language and environment for statistical computing. http://cranr-projectorg.

[47] Schenk J, Rowe K, Steppan S (2013) Ecological opportunity and incumbency in the diversification of repeated continental colonizations by Muroid rodents. Systematic Biology 62: 837–864. 10.1093/sysbio/syt050 23925508

[48] Maddison WP, Maddison DR (2009) MESQUITE: A modular system for evolutionary analysis v 2.75. http://mesquiteprojectorg.

[49] Hasegawa M, Kishino H, Yano T (1989) Estimation of branching dates among primates by molecular clocks of nuclear DNA which slowed down in Hominoidea. Journal of Human Evolution 18: 461–476.

[50] Swofford DL (2012) PAUP*. Phylogenetic analysis using parsimony (*and other methods), v4.0a125 Sunderland: Sinauer Associates.

[51] Revell L (2012) Phytools: An R package for phylogenetic comparative biology (and other things). Methods in Ecology and Evolution 3: 217–223.

[52] Miller MA, Pfeiffer W, Schwartz T (2010) Creating the CIPRES Science Gateway for inference of large phylogenetic trees. Proceedings of the Gateway Computing Environments Workshop (GCE): 1–8.

[53] Duchêne D, Duchêne S (2015) NELSI. 0.2 ed. https://github.com/sebastianduchene/NELSI/blob/master/README.md.

[54] Parham JF, Donoghue PCJ, Bell CJ, Calway TD, Head JJ, Holroyd PA, et al (2012) Best practices for justifying fossil calibrations. Systematic Biology 61: 346–359. 10.1093/sysbio/syr107 22105867 PMC3280042

[55] Magallón S, Castillo A (2009) Angiosperm diversification through time. American Journal of Botany 96: 349–365. 10.3732/ajb.0800060 21628193

[56] Lemmon AR, Brown JM, Stanger-Hall K, Lemmon EM (2009) The effect of ambiguous data on phylogenetic estimates obtained by maximum likelihood and Bayesian inference. Systematic Biology 58: 130–145. 10.1093/sysbio/syp017 20525573 PMC7539334

[57] Marshall CR (2008) A simple method for bracketing absolute divergence times on molecular phylogenies using multiple fossil calibration points. American Naturalist 171: 726–742. 10.1086/587523 18462127

